# Domestication of rice has reduced the occurrence of transposable elements within gene coding regions

**DOI:** 10.1186/s12864-016-3454-z

**Published:** 2017-01-09

**Authors:** Xukai Li, Kai Guo, Xiaobo Zhu, Peng Chen, Ying Li, Guosheng Xie, Lingqiang Wang, Yanting Wang, Staffan Persson, Liangcai Peng

**Affiliations:** 1 Biomass and Bioenergy Research Centre, Huazhong Agricultural University, Wuhan, Hubei 430070 People’s Republic of China; 2 National Key Laboratory of Crop Genetic Improvement, Huazhong Agricultural University, Wuhan, Hubei 430070 People’s Republic of China; 3 College of Plant Science and Technology, Huazhong Agricultural University, Wuhan, Hubei 430070 People’s Republic of China; 4 College of Life Science and Technology, Huazhong Agricultural University, Wuhan, Hubei 430070 People’s Republic of China; 5 School of Biosciences, University of Melbourne, Melbourne, VIC 3010 Australia

**Keywords:** *Oryza*, Transposable elements, Cultivated rice, Wild rice, Evolution

## Abstract

**Background:**

Transposable elements (TEs) are prominent features in many plant genomes, and patterns of TEs in closely related rice species are thus proposed as an ideal model to study TEs roles in the context of plant genome evolution. As TEs may contribute to improved rice growth and grain quality, it is of pivotal significance for worldwide food security and biomass production.

**Results:**

We analyzed three cultivated rice species and their closest five wild relatives for distribution and content of TEs in their genomes. Despite that the three cultivar rice species contained similar copies and more total TEs, their genomes contained much longer TEs as compared to their wild relatives. Notably, TEs were largely depleted from genomic regions that corresponded to genes in the cultivated species, while this was not the case for their wild relatives. Gene ontology and gene homology analyses revealed that while certain genes contained TEs in all the wild species, the closest homologs in the cultivated species were devoid of them. This distribution of TEs is surprising as the cultivated species are more distantly related to each other as compared to their closest wild relative. Hence, cultivated rice species have more similar TE distributions among their genes as compared to their closest wild relatives. We, furthermore, exemplify how genes that are conferring important rice traits can be regulated by TE associations.

**Conclusions:**

This study demonstrate that the cultivation of rice has led to distinct genomic distribution of TEs, and that certain rice traits are closely associated with TE distribution patterns. Hence, the results provide means to better understand TE-dependent rice traits and the potential to genetically engineer rice for better performance.

**Electronic supplementary material:**

The online version of this article (doi:10.1186/s12864-016-3454-z) contains supplementary material, which is available to authorized users.

## Background

Since the discovery of transposable elements (TEs) by Barbara McClintock in maize [1], the origins, roles, and regulation of TEs have been subject to tremendous interest. While TEs are ubiquitous in all organisms [2], their contribution to genome size can change rapidly during evolution. In plants, TEs are defined as two main classes depending on their transposition mechanisms [3]. Class I elements transpose via RNA intermediates, during which the RNA transcripts of a chromosomally integrated copy are used as templates to make DNA (by reverse transcriptase, and RNaseH), and are then inserted into the host genome by the action of integrase or endonuclease components [4]. These TEs are not excised during transposition, which leads to an increase in copy numbers in the host genome. Class I TEs include retrotransposons with long terminal repeats (LTRs), such as Copia, Gypsy and non-LTR retrotransposons. The class II TEs transpose via DNA intermediates, which include Ac/Ds,En/Spm, and Mutator elements, and have mainly been associated with maize [5].

In some species, TEs have caused up to two-fold differences in genome size that sometimes arose over only a few million years [6]. These rapid fluctuations, which may be due to TEs being either more active or more efficiently deleted in different species, indicate that the control of TEs can differ greatly between closely related plant species [7–10]. TEs are often regarded as genomic “parasites” due to the potentially detrimental effects on genes by insertional inactivation and ectopic recombination of DNA [11].

To better understand TE behavior in plants it may be useful to study closely related plant species that span the speciation continuum and that have characteristic biogeographic histories [12]. The *Oryza* clade, consisting of 24 species along an evolutionary gradient of about 15 million years, may work as models for plant genome research and TE evolution [13–16]. Such studies may be well suited to improve rice quality, which is of pivotal significance to worldwide food production and security [13, 14, 17]. Many genes that have led to rice improvement are derived from wild rice AA-genome species, and much attention has been directed on broadening the gene pool of cultivated rice through introgression of other wild relatives of *Oryza* [18]. Phylogenetic analysis of the diploid AA-genome species, i.e., *O. sativa* Japonica, *O. sativa* Indica, *O. nivara*, *O. rufipogon*, *O. glaberrima*, *O. barthii*, *O. glumaepatula* and *O. meridionalis*, indicated that a closely spaced series of recent speciation events in this genus has occurred [19]. These species span a wide range of global pantropical geographical regions and are disjunctively distributed in Asia, Africa, Australia, and South America [20]. Having diverged approx. 3 Mya from a common AA-genome ancestor [19], these eight species have contributed extensive adaptive and breeding traits [21, 22].

The *Oryza* AA-genome species contains eight diploid species, including two cultivated species *Oryza sativa* L. ssp. japonica and *Oryza glamberrima* Steud. [20]. In general, *O. sativa* is grown in China and other Asia-Pacific regions, and has two subspecies, *O. sativa* Japonica and *O. sativa* L. ssp. indica [23]. By comparison, *O. sativa* Japonica is an important model species of monocot plants and cereals [24]. *Oryza rufipogon* Griff. and *Oryza nivara* are widely recognized as the direct ancestors of Asian cultivated rice (*O. sativa*) [21, 25–30]. In particular, *O. rufipogon* is perennial, photoperiod sensitive, largely cross-fertilized and widely distributed from the Southern China, South and Southeast Asia to Papua New Guinea and Northern Australia. It grows perennially in areas around water such as swamps and lakes [31]. In contrast, as a wild rice from India, *O. nivara* is an annual, photoperiod insensitive and predominantly self-fertilized species. This species is restricted to South, and mainland Southeast, Asia in the diverse areas such as swampy lands, edges of ponds and tanks, beside streams, ditches, in or around rice fields [32, 33]. *O. glaberrima*, a West African species of cultivated rice, was independently domesticated about 3000 years ago in the Niger River Delta. It has significant resistance to many pests and diseases and tolerance to drought and poor soils, but lacks many of important agronomic traits compared with Asian rice [34]. Furthermore, *Oryza longistaminata* and *Oryza barthii* are the progenitors of *O. glaberrima* [35]. *O. barthii* is normally found in mopane, savanna woodland, savanna and fadama, and prefers to grow in clay or black cotton soils [24]. *Oryza glumaepatula* and *Oryza meridionalis* are the AA-genome diploid wild rice found in Latin America and Australia, respectively. *O. glumaepatula* grows in deep and sometimes flowing water, whereas *O. meridionalis* is found at edges of freshwater lagoons, temporary pools, and swamps in 15–20 cm of water [24].

In this study, we used these closely related rice species to map the TE distributions in their genomes. We found that TEs are depleted from genomic regions that are associated with genes in cultivated rice species. In addition, common genes and gene families are associated with, or devoid of, TEs in the cultivated species, suggesting that domestication has affected TE distribution similarly in these species despite a relatively distant evolutionary history.

## Results

### Evolutionary relationships of eight rice AA genomes

**Fig. 1:**
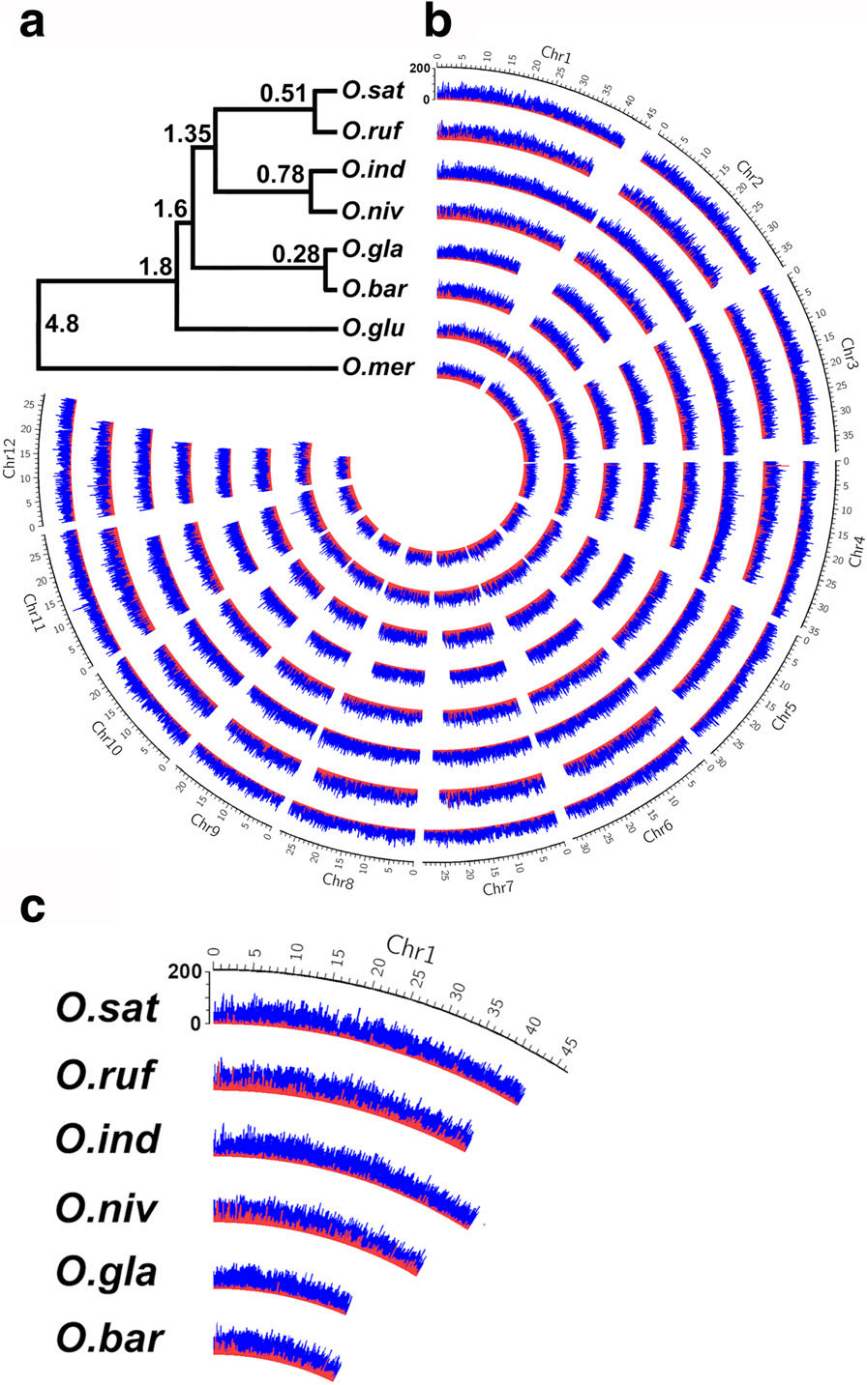
Comparative genomics of the eight AA-genome *Oryza* species. **a** Phylogenetic relationship of the eight AA-genome *Oryza* species inferred from orthologous gene sequences. Estimates of divergence time (million years) are given at each node (6.5 × 10^−9^ substitutions per site per year), all supported with 100% bootstrap values. **b** Comparative genome analysis. Each line in the circle represents one of the 12 chromosomes of *Oryza* genomes, along with the number of all TEs (*blue*) and TEs in genes (*red*). And the slide window size is 100,000 bp. **c** Blown-up image of Chr1 for six of the rice species

To confirm the evolutionary relationship between the eight rice species, we performed phylogenomic analyses of 1937 orthologous and single-copy genes of at least 300 aa from the eight fully sequenced *Oryza* genomes, using *O. meridionalis* as out-group (Fig. 1a and Additional file 1: Table S1). Gene-based phylogenetic relationships clearly supported global genome divergences of the species. The general nucleotide substitution rate has been estimated to 6.5 × 10^−9^ substitutions per site per year [36], which we used to date the speciation events. We found the major extant AA lineages to be within 4.8 million years (Fig. 1a), in agreement with other analyses of sequence divergence times for rice [36].

### TEs are larger and occupy more genomic space in cultivated rice as compared to wild relatives

**Fig. 2:**
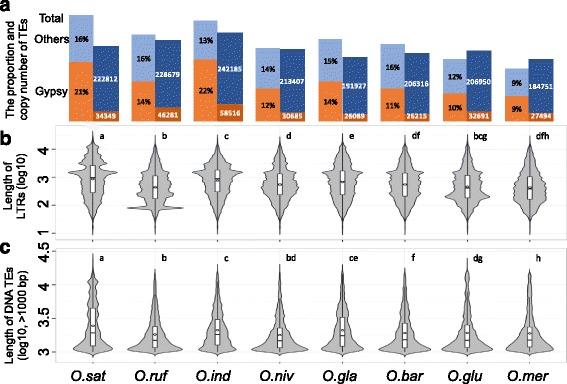
TEs occupy more space in cultivated rice genomes as compared to their wild relatives. **a** The proportion and copy number of Gypsy and Total TEs in the different rice genomes. The left bars represent relative amounts of Gypsy and Total TE sizes compared to the genome sizes, and the right bars represent the number of Gypsy and Total transposons in the respective genomes. **b**-**c** Violin graphs of the length of LTR transposons (**b**), and DNA transposons (**c**), in the different rice genomes. The *violin bars* followed by the same letter are not significantly different (*P* < 0.05) as determined by Student’s *t* test

Cultivated rice cultivars have larger genome sequences, and typically have more genes and transcripts, as compared to their closest wild relatives [37–41] (Table 1). To investigate whether the number and sizes of TEs vary among these species we employed a homology-based search of a TE library as query sequences. More specifically, we used RepeatMasker to perform *de novo* homology search for TEs of the rice genomes (Repbase 20140131 [42], with parameter: -e rmblast -gff –species rice -nolow -norna -xsmall). We refer to the obtained segments as TEs, but it is noteworthy that many of them were short TE fragments. Figures 1 and 2 show the distribution, and the length and copy numbers of TEs in the rice genomes, respectively. These data revealed that 18.4 to 37.5% of the genome sequences consist of TEs, which corresponds to approximately 0.2 million TEs (Additional file 2: Table S2; Additional file 3: Table S3). Notably, 37.5, 30.5, 35.5, 25.7, 28.9, 27.1, 21.9 and 18.4% of the genomes of *O. sativa* Japonica, *O. rufipogon*, *O. sativa* Indica, *O. nivara*, *O. glaberrima*, *O. barthii*, *O. glumaepatula* and *O. meridionalis*, consisted of TEs, respectively (Fig. 1b; Additional file 2: Table S2), indicating that a relatively larger fraction of the genomes of the three cultivated rice species consisted of TEs as compared to their close wild relatives. The majority of these were LTR elements, followed by DNA transposons and LINEs (Additional file 2: Table S2). The LTR elements contributed 55 to 104 Mbp (or 17 to 25%) of the cultivated rice genomes, while only 38 to 58 Mbp (or 11 to 17%) of the wild rice genomes. A complete list of the contribution of different TE classes to the genomic TE content is outlined in the supplemental material (Additional file 2: Table S2; Additional file 3: Table S3).

**Table 1:** The sequenced genomes of the eight *Oryza* species in this study

Species	Geographical origin	Category	Sequence length	Genes^a^	Transcripts^a^
*Oryza sativa* Japonica	All over the world	Cultivated	374471240	89669	114289
*Oryza rufipogon*	Asian, India and Australia	Wild	338040714	63530	100603
*Oryza sativa* Indica	Southern Asia	Cultivated	411710190	86322^b^	88438^b^
*Oryza nivara*	Asian and India	Wild	337950324	58613	98640
*Oryza glaberrima*	African	Cultivated	316419574	78722	103316
*Oryza barthii*	West African	Wild	308272304	56968	88012
*Oryza glumaepatula*	South America	Wild	372860283	60679	99411
*Oryza meridionalis*	Australia	Wild	335668232	51551	87383

^a^RNA-seq supported criterion is based on Cufflinks predictions, and include coding and non-coding genes

^b^No RNA-seq to support genes/transcripts, data source is from http://plants.ensembl.org/Oryza_indica/Info/Annotation

The TEs also contributed larger blocks of sequence in cultivated rice than that in their wild relatives (Fig. 2). For example, Gypsy LTR elements were typically two times longer in sequence in cultivated Asian rice species as compared to their closest wild rice species (Fig. 2b and Additional file 2: Table S2). However, the copy number of these specific TEs did not strictly follow this trend (Fig. 2a; for example, 34349, 46281, 58516 and 30685 copies of Gypsy for *O. sativa* Japonica, *O. rufipogon*, *O. sativa* Indica and *O. nivara*, respectively). Nevertheless, our results indicated that TEs contribute a larger part of the genome sequences, and were longer in sequence, in cultivated rice species as compared to their close wild relatives (*χ*2 Fisher’s exact test; *P* value ≤ 0.01 for each pair).

### TEs are selectively excluded from gene regions in cultivated rice

**Fig. 3:**
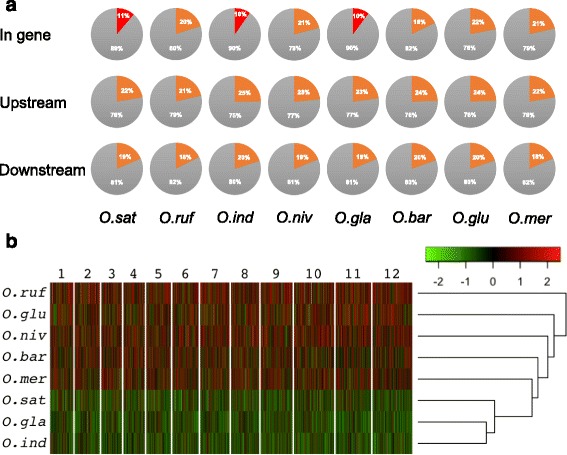
Gene regions contain lower amounts of TEs as compared to their wild relatives. **a** Pie charts showing the relative distributions of TEs associated with genes, and upstream and downstream gene regions. Note that the gene regions in the domesticated species contain relatively less TEs as compared to their wild relatives. Such differences are not evident in the upstream and downstream regions of the genes. **b** *k*-means clustering with heatmap based on the distribution of TEs in genes of the 12 chromosomes of the *Oryza* genomes. The color scale represents the number per 100,000 bases of TEs in genes (*green, black* and *red* refer to low, medium and high TEs numbers, respectively)

To assess whether the TEs were associated with specific genomic regions, we analyzed whether there were any differences in TE distribution among genes in the rice genomes. We defined a gene as a continuous exon and intron sequence, including the predicted surrounding untranslated regions. We mapped the TEs onto the rice genomes and observed that at least 10% of TEs occurred in such gene regions (Fig. 3a, Additional file 4: Figure S1 and Additional file 3: Table S3). Surprisingly, the three cultivated rice species contained substantially lower numbers of TEs associated with genes as compared to their closest wild rice species (Fig. 3, Additional file 4: Figure S1 and Additional file 3: Table S3). While the cultivated species only contained 9.7 to 11.2% of the total TEs inserted in gene regions, the related wild relatives contained 17.8 to 21.9% of their TEs in genes (Fig. 3 and Additional file 3: Table S3). For example, of the total Gypsy TEs only 3.2 to 6.0% of the copies were in gene regions of the cultivated rice species while between 9.5 to 14.2% of the total Gypsy TE copies were in corresponding regions of the wild rice species (Additional file 3: Table S3). We found that the major TEs in the gene regions were DNA transposons, while TEs in intergenic regions were often LTR retrotransposons. Moreover, we also counted the numbers of TEs within 2 kbps upstream and downstream of the genes (Fig. 3a). Notably, these regions did not contain any substantial differences in TEs, i.e., similar levels of TEs were found in these regions both in the cultivated and in the wild rice species (Additional file 5: Table S4; Additional file 6: Table S5). Nevertheless, some TEs, including the LTR TEs, showed tendencies to occupy these areas differently in the different rice species. To estimate whether the TE content is lower in gene regions than expected by chance we compared the observed numbers with random TE occupations based on the TE content in the respective rice genomes. We found strong significant support for lower levels of TEs associated with genes in the cultivated vs wild rice species (Test for equal or given proportions; *P* value ≤ 1.9E-06; Additional file 7: Table S6). These findings indicate that the cultivated rice species deprive TEs from their gene regions, and suggest that TEs may promote certain environmental adaptations of genes in their wild rice relatives.

### TEs are preferentially present in introns of expressed genes

**Fig. 4:**
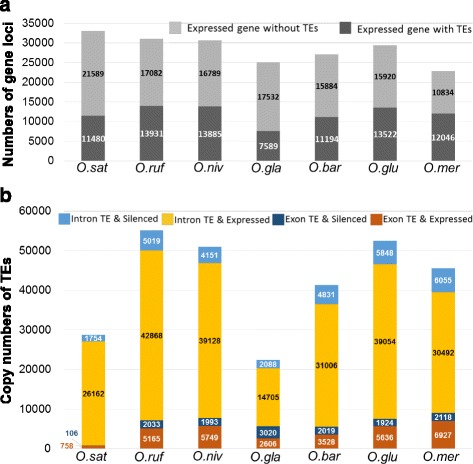
TEs are preferentially located to intronic gene regions. **a** Expressed genes with or without TEs. **b** Numbers of TEs in expressed or not expressed genes based on RNA-seq transcriptomic data

To investigate how the TEs affected gene functions, we examined gene expression levels by Cufflinks using FPKM (fragments per kilobase of exon per million fragments mapped) from RNA sequencing data (RNA-seq; CPGS *Oryza* Genome Evolution Project, https://www.nsf.gov/awardsearch/showAward?AWD_ID=1026200) of seven of the eight rice species (exception; *O. sativa* Indica as no data was available). We defined a gene with FPKM values greater than zero as expressed, otherwise the gene was referred to as non-expressed. The RNA-seq data were obtained from leaves, roots and panicles, and many genes will therefore not be called expressed due to tissue/cell type specific expression. We took note of this during our subsequent analyses and for our conclusions. As a result, 33069, 31013, 30674, 25121, 27078, 29442, and 22880 of the genes in *O. sativa* Japonica, *O. rufipogon*, *O. nivara*, *O. glaberrima*, *O. barthii*, *O. glumaepatula* and *O. meridionalis*, respectively, was referred to as expressed in leaves, panicles and roots (Fig. 4a). We next assessed whether these genes were associated with TEs. We found that 11480, 13931, 13885, 7589, 11194, 13522, 12046 of the expressed genes have TEs associated with the gene regions in *O. sativa* Japonica, *O. rufipogon*, *O. nivara*, *O. glaberrima*, *O. barthii*, *O. glumaepatula* and *O. meridionalis*, respectively (Fig. 4a). Notably, we observed that expressed genes typically contain TEs in their introns rather than in exons (Fig. 4b). For example, *O. sat* Japonica only has 758 TEs in exons, whereas 26162 TEs are in introns, of its expressed genes (Fig. 4b). This is almost a 34-fold difference in TE distribution within the gene regions of expressed genes, and is substantially more than what is expected by chance (*χ*2 > 8000; *P* value of all species are zero). In contrast, the numbers of TEs in introns or exons were almost equal for the genes referred to as non-expressed (Fig. 4b). These data indicate that, at least in leaves, panicles and roots of rice species, intronic rather than exonic TEs are associated with expressed genes. These data support that TEs are an unwanted feature in exons that may cause deleterious effects on gene function and/or expression.

### TEs have been selectively retained in genes associated with certain functions in cultivated rice species

To investigate if the TEs preferentially affected genes of certain functions, we performed Gene Ontology (GO) enrichment analyses (Additional file 8: Table S7). We first performed GO analyses for genes that contain TEs in their promoter regions (2 kbp upstream of gene), within the gene coding regions and 2 kbp downstream for the rice species (Additional file 8: Table S7). Interestingly, different sets of GO terms were significantly enriched for TEs in the three regions for cultivated vs wild rice species, respectively. That is, the three cultivated species *O. sativa*, *O. indica* and *O. glaberrima* all contained a very similar set of GO terms that were affected by TEs, e.g., GO terms such as “cell wall”, “flower development” and “cell death” (Additional file 8: Table S7). Likewise, the wild rice species displayed their own set of GO terms affected by TEs, including the GO terms “response to biotic stimuli” and “kinase activity”. We next assessed whether genes, and upstream and downstream gene regions, that are devoid of TEs also showed similar GOs among the cultivated vs. wild rice species, respectively. Indeed, also here we found that the cultivated rice species had common GO terms significantly enriched, including the terms “plastids” and “thylakoids” (Additional file 8: Table S7). Likewise, the wild rice species also had similar GO terms enriched among themselves for the investigated genomic regions, e.g., “response to endogenous stimulus” and “chromatin binding”. These data indicate that TEs have become enriched (or devoid) in genes of certain functions depending on domestication.

**Fig. 5:**
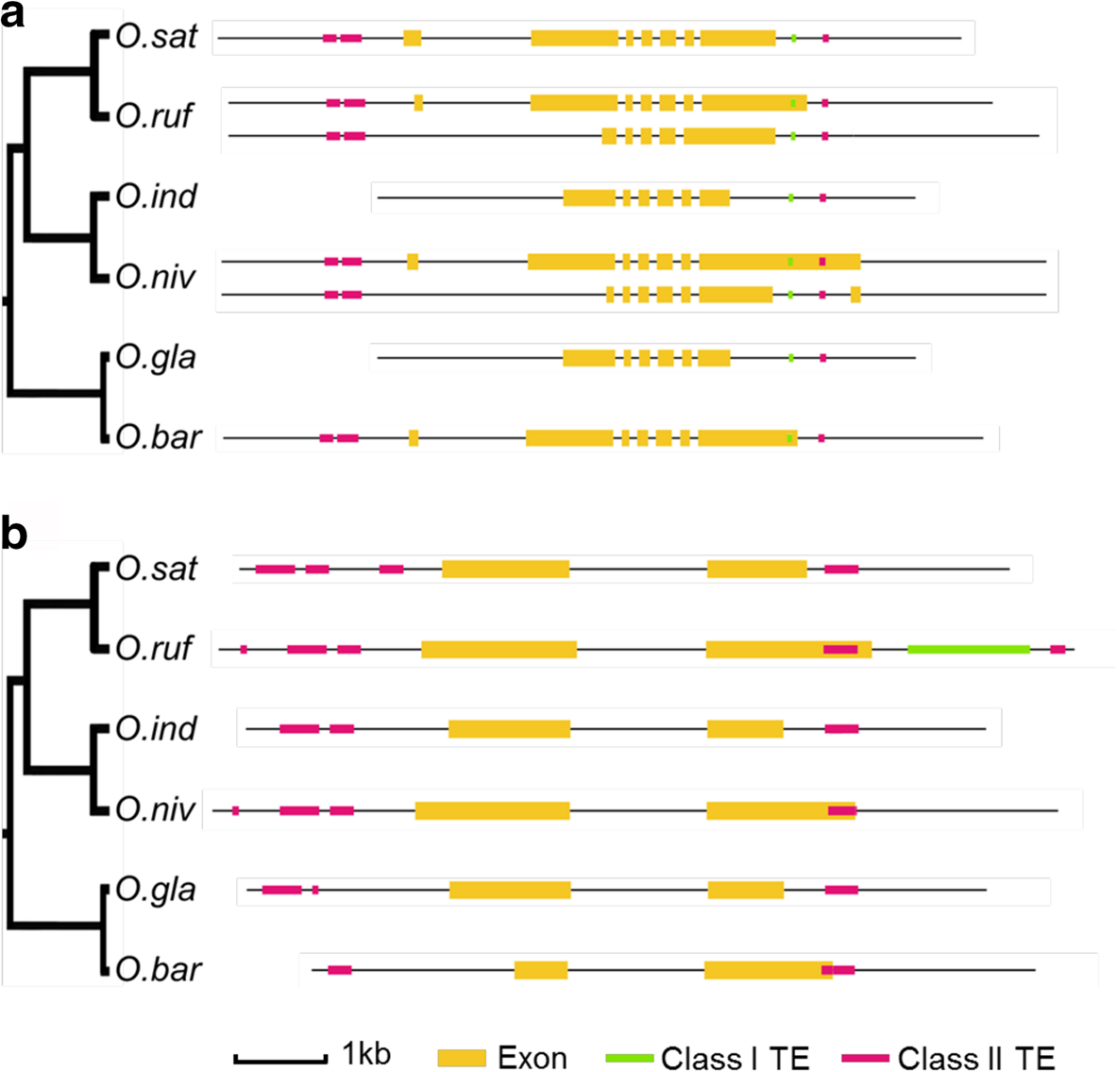
Pair-wise comparisons of gene structure and TE locations of two examples within GO:0016301. **a** LOC_Os07g48290 gene. **b** LOC_Os02g45750 gene

We next assessed whether the genes in the enriched GO terms are close homologs, i.e., if the genes included in a certain GO term are closely related genes for the cultivated rice vs their wild relatives. Surprisingly, we found that many of the genes associated with any given enriched GO term represented close homologs across the cultivated rice species. For example, TEs were absent in the last exon region of closely related genes in cultivated rice, but present in wild rice, of the GO term GO:0016301 (kinase activity; Fig. 5). Two such examples are illustrated in Fig. 5, in which gene coding regions (exon) of close gene homologs in wild rice (*O. rufipogon*, *O. nivara* and *O. barthii*) contained TEs (class I TEs in Fig. 5a; class II TEs in Fig. 5b), whereas the closest homologs in the cultivated rice species (*O. sativa* Japonica, *O. sativa* Indica and *O. glaberrima*) did not. Since the wild rice species have a pairwise evolutionary relationship with the cultivated species, i.e., the *O. sativa* Japonica is more closely related to *O. rufipogon* than to *O. sativa* Indica (Fig. 1), these data suggest either a similar change in the coding regions of genes in the cultivated rice species (as exemplified in Fig. 5), or an independent loss or gain of the TEs in the cultivated and wild rice species, respectively. Notably, the gene structures are supported by RNA-seq data (Additional file 9: Figure S2). Nevertheless, the domestication appears to have led to convergent evolution of coding regions of genes, and/or TE content and positions in rice species.

### TEs may affect gene functions that are central to growth and traits differently in cultivated vs wild rice species

Emergence, extinction and alterations of genes within closely related organisms can lead to species adaptation and lineage evolution [43]. To assess whether the TEs may contribute to important traits in cultivated rice, we first assigned *O. sativa* Japonica, *O. sativa* Indica, *O. nivara*, *O. rufipogon*, *O. glaberrima*, *O. barthii*, *O. glumaepatula* and *O. meridionalis* protein sequences to OrthoMCL families, and identified 31,860 orthologous gene families (except singletons) comprising 393683 genes. We compared the Asian (i.e., *O. sativa* Japonica and Indica) and African (*O. glaberrima*) cultivated rice to their closest ancestor wild rice, and analyzed the location of TEs in gene families associated with important traits in peer-reviewed publications. We found that both regulatory proteins (e.g., PF01486-MADS-box family, and PF00646-F-box protein) and metabolic proteins (e.g., PF0832-GT5, PF03254-xyloglucan fucosyltransferase, PF04616-GH43, etc.) had much reduced numbers of TEs in their gene coding regions in cultivated rice species as compared to their wild relatives (Table 2). The corresponding proteins may partake in starch (PF08323), cell wall hemicellulose (PF03254) synthesis, storage (PF01734) and seed imbibition (PF05691) processes, or be involved in chloroplast carboxylation (PF00194) and sugar transport (PF03083). Other protein families were associated with biotic or abiotic stress tolerance (PF00743) or signalling pathways (PF00141). In summary, protein families that are important for rice development and growth are affected by TEs differently in cultivated and wild rice species.

**Table 2:** Examples of gene families that are known to be associated with traits in rice and their TEs insertions numbers

PFAM^a^		Cultivated rice									Wild rice								
		*O.sat*			*O.ind*			*O.gla*			*O.ruf*			*O.niv*			*O.bar*		
PF08323	Glycosyl Transferaes family 5	13^b^	8^c^	21^d^	9	8	16	9	11	17	12	15	19	14	15	15	17	12	17
PF00646	F-box protein	56	21	51	56	29	48	33	4	22	26	153	26	23	113	24	27	106	17
PF03254	Xyloglucan fucosyltransferase	21	6	17	15	12	19	18	7	15	5	43	3	3	41	2	1	46	1
PF00194	Carbonic anhydrase	4	2	1	0	1	1	2	1	2	6	19	2	4	33	3	2	15	3
PF04616	Glycoside Hydrolase family 43	2	1	3	6	5	4	4	1	2	4	7	2	4	7	2	7	6	3
PF01486	MADS-box family	0	0	1	1	0	1	0	0	1	2	4	1	2	4	1	2	4	1
PF01734	patatin protein family	3	5	9	9	5	8	4	3	6	7	21	4	9	34	3	5	12	3
PF03083	Flavin-containing monooxygenase	2	3	1	3	1	2	2	1	2	2	11	0	3	7	0	5	8	1
PF00743	Flavin-containing monooxygenase	10	2	3	8	1	3	1	1	0	4	18	1	4	14	1	3	12	0
PF05691	Glycoside hydrolase family 36	7	2	9	11	2	9	7	2	9	7	4	9	9	6	7	7	3	9
PF00141	Haem peroxidase	1	0	1	4	0	1	0	0	1	2	5	1	4	5	3	3	5	2

^a^The PFAM is a large collection of protein families, each represented by multiple sequence alignments and hidden Markov models

TEs insertions numbers in

^b^2kb upstream

^c^gene regions and

^d^2kb downstream of a gene

**Fig. 6:**
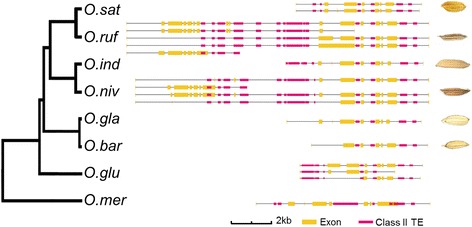
Comparisons of gene structure and TE locations of *GIF1* gene critical for grain filling in the eight rice species. Organization of exons, introns and TEs of *GIF1* (*GRAIN INCOMPLETE FILLING 1*; LOC_Os04g33740) gene in gene body and 2-kbp flanking sequences of the gene. Seed images of ancestral wild rice and cultivated rice are shown to the *right*

To illustrate how the TEs might affect specific genes that have been shown to directly affect important rice traits, we inspected several gene regions related to grain development. The rice cell-wall invertase (CWI) gene *GIF1* (*GRAIN INCOMPLETE FILLING 1*) controls grain-filling, yield [44] and seed size [45] in rice, and domestication might have changed its promoter region that affected *GIF1* expression [46]. We found that both the gene coding region and the promoter (2 kb upstream) contained TEs in the cultivated and the wild rice species (Fig. 6). Notably, sequence alignment and annotation of the orthologous genomic regions for the eight rice species revealed conserved gene colinearity and structure in the *GIF1* region, but the TE content and positions clearly differed (Fig. 6). This includes many Class II TEs that were present in the promoter regions of the *GIF1* in the wild rice relatives. These TEs might therefore affect either alternative splicing or changes in expression of the *GIF1* locus, which may contribute to the grain-filling capacity in the rice species.

**Fig. 7:**
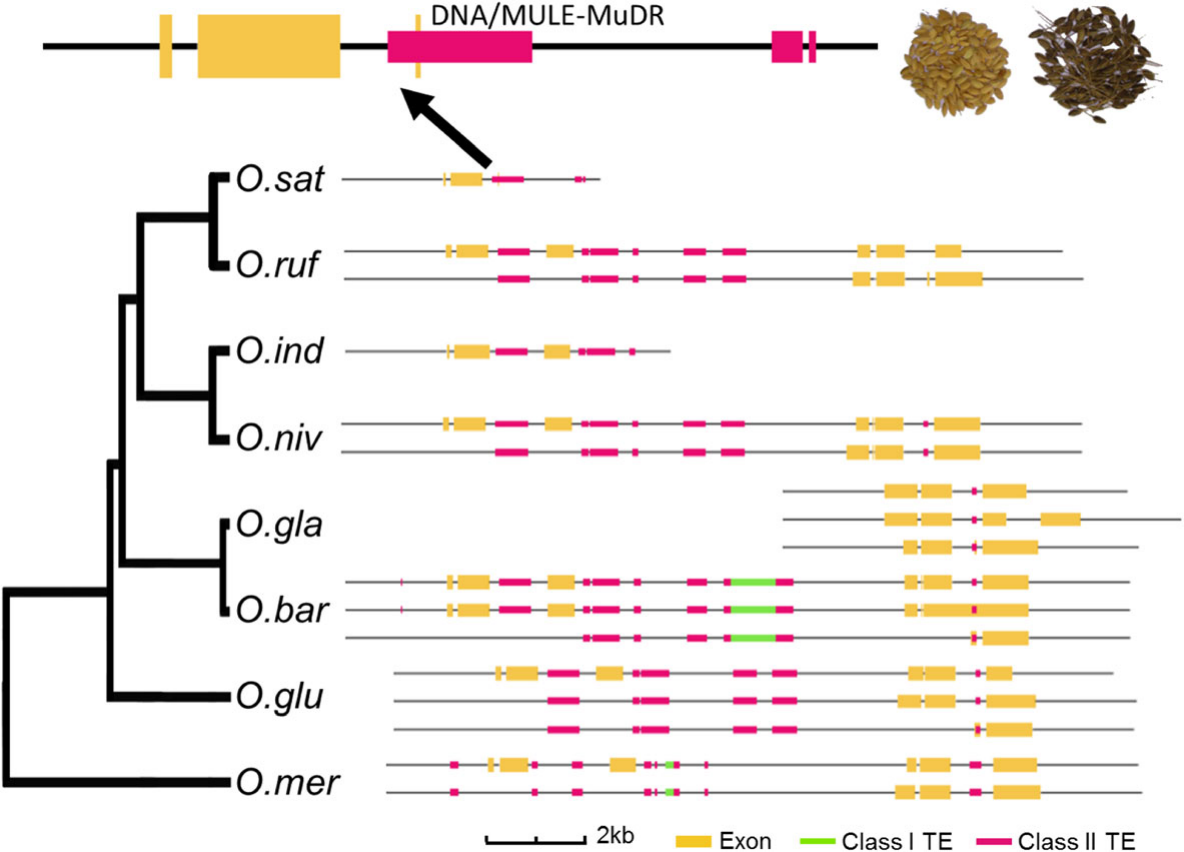
Comparison of gene structure and TE locations of *BH4* gene critical for grain filling in the eight rice species. Organization of exons, introns and TEs of *BH4* (*BLACK HULL4*; LOC_Os04g38660) gene in gene body and 2-kbp flanking sequences of the gene. A blown-up image of *BH4* gene structure of *O. sativa* Japonica is shown above the main image. This mutation changed the black-colored seed hull of the ancestral wild rice to the straw-white seed hull of cultivated rice

Another gene that is important for seed development in rice is the *BH4* (*Black Hull4*) [47], which encodes an amino acid transporter. This gene is key to explain the transition from the black-colored seed hull of the ancestral wild rice to the straw-white seed hull of cultivated rice during grain ripening [47]. The straw-white hull was selected as an important visual phenotype of non-shattered grains during rice domestication. A 22-bp deletion within exon 3 of the *BH4* disrupted the gene function, leading to straw-white hull in several cultivated rice species [47]. Interestingly, this exon is affected by a MULE-MuDR DNA transposon in *O. sativa* that must have caused an alternative splice variant of *BH4* in this rice species (Fig. 7). In *O. sativa* Japonica, part of the MULE-MuDR was transcribed as exon 3 (24 bp), although the other seven rice species also had this MULE-MuDR, transcription bypassed this transposon (Fig. 7). Nevertheless, these data support an important role of TEs in the selection of rice species during domestication.

## Discussion

TEs are important genomic components that may underpin rapid evolutionary processes in various organisms [48–50]. Using three cultivated and their close wild rice species, we have delineated the genomic content and distribution of TEs, which revealed that TEs tend to be devoid from gene coding regions and affect different sets of genes in the cultivated species despite a more distant evolutionary relationship compared to their close wild rice relatives.

Our study reveals two major and surprising findings; 1. Gene regions in the cultivated species are devoid of TEs, and 2. Genes of certain functions tend to be similarly affected by TEs in cultivated species, but different in wild rice species. It is plausible that the cultivated species have been selected to maintain certain features, including stable expression of trait-related genes, and that this is the underlying factor behind the TEs distributions. Indeed, we found that the wild rice species contained more TEs in their gene regions, mainly associated with intronic regions, which could affect expression levels and alternative splicing of genes that may be detrimental to important traits in cultivated species. For instance, many TE mutations are harmful to seed yields [51]. On the other hand, an association of TEs with the gene regions, as that observed in the wild rice species, might allow for plasticity of gene expression and could perhaps also affect chromatin features, including euchromatin and heterochromatin regions [48, 52]. In contrast to the gene regions, we found that both cultivated and wild rice species have similar copy numbers of TEs in their 0-2kbp upstream, or downstream of genes, indicating that these TEs may not have been as affected during domestication/selection of cultivated rice, and perhaps affect gene functions less than their gene-located counterparts. Interestingly, in at least some of the cases where we investigated how certain TEs affected loci in more detail, we found that the coding regions sometimes had changed in a converging fashion in the cultivated species. These changes could to some degree also explain why the gene regions in our analyses were associated with fewer TEs in these species. While we confirmed the changed gene structures with RNA-seq data, it is noteworthy that the cultivated species are better annotated as compared to the wild rice species and so some differences in gene coding regions may also be due to poor annotation.

The fact that TEs affected genes that are closely related differently in the cultivated vs the wild rice species underscores the importance of maintaining certain functions stable in the cultivated species [53]. The most likely scenario for why this has occurred may be that the domestication and subsequent selection of rice has slowly led to depletion of TEs from genes that underpin important traits in rice. We exemplified some of these genes, for example the *GIF1* locus, which support our hypothesis that the TEs may influence loci that encode important rice traits [44–46]. The similar TE distributions in cultivated rice may therefore be a domesticated evolutionary convergence of TE locations [54]. In other words, we interpret that the domestication process of rice has primarily affected the genomic distribution of TEs, and that certain rice traits are better maintained based on the TE distribution patterns. Hence, our findings have provided insights into TE-dependent rice traits and suggest potential strategies for genetically engineering of rice.

It is important to note that because we have analyzed one accession per species (a typical way to perform genomic studies) it might be difficult to conclude how many TEs are truly missing and/or present in any of the species. However, we assume that the accessions we chose are representative for the different species. Nevertheless, any minor discrepancies in TE distribution based on this would not impact on the overall message of this study. Hence, cultivation of rice has led to a general TE depletion from genes and to a selection-based evolutionary convergence in terms of gene functions affected by TEs.

## Conclusions

TEs are important genomic landmarks of genetic variation, and different types of TEs, as well as their physical location and abundance, provide for insights into genome evolution. This study has demonstrated that domestication of rice has primarily impacted the genomic distribution of TEs and that certain rice traits are closely associated with TE distribution patterns. The results have provided insights into TE-dependent rice traits and indicated the potential to genetically engineer rice for high seed yields and biomass production.

## Methods

### Annotation of TEs in eight *Oryza* genomes

The eight *Oryza* genome sequence and annotation data were downloaded from the Ensembl Plants release 27 (ftp://ftp.ensemblgenomes.org/pub/plants/) [37] FTP site (ftp://ftp.ensemblgenomes.org/pub/plants/). RepeatMasker (version 4.0.5 with repbase 20140131) [55] was used to *de novo* search for TEs in the *Oryza* genomes (-e rmblast -gff –species rice -nolow -norna -xsmall), including repetitive elements, such as simple repeats, satellite DNAs, SINEs, LINEs and DNA transposons. It should be noted that the annotation of the RepeatMasker cannot be directly used to detect full-length LTR retrotransposons because they lack information on which LTR belongs to which LTR retrotransposon. A full-length LTR retrotransposable element means an element that has two LTRs at each ends. Full-length LTR retrotransposons and their exact positions were investigated using two software programs, LTRharvest [56] and LTR_FINDER [57] with default settings. These programs are designed to identify full-length LTR retrotransposons that possess a pair of high homology regions but use different algorithms.

### Estimation of genome divergence

The global extent of genome divergence between Asian cultivated rice (*O. sat*) and the other 7 AA-genome Oryza was analyzed by gKaKs [58]. The average synonymous substitutions (dN) exceeded nonsynonmous substitutions (dN) for each pairwise comparison, suggesting a widespread purifying selection of detected orthologous genes. Both synonymous and nonsynonmous substitutions were in agreement with their positions in the phylogenetic topology, indicating an increase of substitutions with species divergence.

### RNA-seq, mapping and transcript assembly

The leaf, panicle and root RNA-seq raw fastq files were downloaded for 7 *Oryza* species from the NCBI SRA (http://www.ncbi.nlm.nih.gov/sra): *O. sativa* japonica (SRX477950, SRX477951, SRX477952), *O. nivara* (SRX472708, SRX472710, SRX472709), *O. rufipogon* (SRX512340, SRX512341, SRX512342), *O. glaberrima* (SRX474528, SRX474529, SRX474530), *O. barthii* (SRX471823, SRX472434, SRX472435), *O. glumipatula* (SRX475002, SRX475003, SRX475004), *O. meridionalis* (SRX475006, SRX475007, SRX475008) from NCBI SRA [59]. We mapped the reads against the respective reference genome using Tophat [60], allowing up to two mismatches and discarding reads mapping at multiple locations. The TopHat’s read alignments were assembled by Cufflinks and its associated utility program to produce transcriptome annotation and gene expression of the genomes [61].

### Orthologous gene pairs and gene colinearity

Whole-genome protein sequences of eight *Oryza* were merged and searched against each other for homology using BLASTP [62] with an E-value cutoff of 10^−5^ and parsed out top five hits. The core-orthologous genes were defined by OrthoMCL [63]. The colinearity blocks between two *Oryza* species were calculated by software MCScanX with default parameters [64].

### GO enrichment analysis

GO terms associated with genes of the cultivated and wild rice species were used to perform enrichment analysis of genes with or without TEs, including gene regions, 0–2 kb upstream and downstream regions. For example, given a set of genes that contains TEs in the upstream gene region, an enrichment analysis will find which GO terms are over-represented using annotations for that gene set. Commonly enriched GO terms for the three cultivated rice or their ancestors were then identified and highlighted (Additional file 8: Table S7). A list the genes with conserved synteny regions, which are significantly enriched for eight GO terms in three species, is include in Additional file 8: Table S7.

### Statistical analysis

To estimate whether the cultivated rice TEs content is lower than their wild ancestor rice, we used a test for equal or given proportions by R. It can be used for testing the null hypothesis that the proportions in several groups are the same, or that they are equal given the starting values. For the comparative analysis of exonization levels, we used a contingency table Chi-square test (*χ*2 test). When the contingency table was a 2 × 2 table, the Fisher’s exact test was used. Data analyses were performed using the statistical R package.

## Additional files

Additional file 1: Table S1. Genomic divergence between *O. sativa* Japonica and the other 7 *Oryza* species by genome-level Ka/Ks calculation. (PDF 106 kb)
Additional file 2: Table S2. Coverage (Mbp) and proportion of TE insertion in *Oryza*. (PDF 30 kb)
Additional file 3: Table S3. Transposon insertions in the Genomes, and in the gene regions. (PDF 30 kb)
Additional file 4: Figure S1. TEs location in 2384 genes obtained from conserved synteny regions via *Oryza* transcriptome data. (PDF 133 kb)
Additional file 5: Table S4. Transposon insertions in the Genomes, and in the 0-2 kb regions upstream of a gene. (PDF 30 kb)
Additional file 6: Table S5. Transposon insertions in Genome and 0-2 kb downstream of a gene. (PDF 31 kb)
Additional file 7: Table S6. Statistical table of transposon insertions in Genome and gene. (PDF 252 kb)
Additional file 8: Table S7. Gene Ontology terms associated with genes enriched or devoid of TEs in cultivated and wild rice species. (XLS 204 kb)
Additional file 9: Figure S2. The gene structures are supported by RNA-seq data. (A) LOC_Os07g48290 gene. (B) LOC_Os02g45750 gene. (PDF 27 kb)

## Abbreviations

BLAST: Basic local alignment search tool
bp: base pair
CWI: Cell-wall invertase
dN: The number of non-synonymous substitutions per non-synonymous site
dS: The number of synonymous substitutions per synonymous site
FPKM: Fragments per kilobase of exon per million fragments mapped
GO: Gene ontology
LINEs: Long interspersed nuclear elements
LTRs: Long terminal repeats
Mbp: Mega base pairs
Mya: Million years ago
NCBI: National Center for Biotechnology Information
RNaseH: Ribonuclease H
RNA-seq: RNA sequencing
SINEs: Short interspersed nuclear elements
SRA: Sequence read archive
TEs/TE: Transposable elements
UTR: Untranslated region, including the 5’ and 3’ UTRs
