# Supplementary material for: Domestication of rice has reduced the occurrence of transposable elements within gene coding regions
Source: BMC Genomics. 2017 Jan 9;18:55. doi: 10.1186/s12864-016-3454-z (PMC5223533; doi:10.1186/s12864-016-3454-z)
Supplement: Additional file 1: Table S1. — Genomic divergence between O. sativa Japonica and the other 7 Oryza species by genome-level Ka/Ks calculation. (PDF 106 kb) [file 12864_2016_3454_MOESM1_ESM.pdf]

Supplementary table S1. Genomic divergence between *O. sativa* Japonica and the other 7 *Oryza* species by genome-level Ka/Ks calculation

**Pairwise Species**	**Orthologous Singletons**		
	**dN**	**dS**	**ω**
O.sat – O.ruf	0.009	0.026	0.833
O.sat – O.ind	0.009	0.025	0.740
O.sat – O.niv	0.013	0.039	0.661
O.sat – O.bar	0.015	0.048	0.643
O.sat – O.gla	0.016	0.049	0.641
O.sat – O.glu	0.017	0.049	0.591
O.sat – O.mer	0.025	0.093	0.491

Note: dN: non-synonymous substitutions; dS: synonmous substitutions.
