# Supplementary material for: Domestication of rice has reduced the occurrence of transposable elements within gene coding regions
Source: BMC Genomics. 2017 Jan 9;18:55. doi: 10.1186/s12864-016-3454-z (PMC5223533; doi:10.1186/s12864-016-3454-z)
Supplement: Additional file 2: Table S2. — Coverage (Mbp) and proportion of TE insertion in Oryza. (PDF 30 kb) [file 12864_2016_3454_MOESM2_ESM.pdf]

Supplementary table S2. Coverage (Mbp) and proportion of TE insertion in *Oryza*

Repeat family	*O.sat*	*O.ruf*	*O.ind*	*O.niv*	*O.gla*	*O.bar*	*O.glu*	*O.mer*
**Retroelements**								
SINEs:	1.3[a]	1.2	1.3	1.2	1.1	1.2	1.2	1.1
	0.4%[b]	0.4%	0.3%	0.4%	0.3%	0.4%	0.3%	0.3%
LINEs:	4.1	3.7	4.2	3.7	3.5	3.6	3.5	2.9
	1.1%	1.1%	1.0%	1.1%	1.1%	1.2%	0.9%	0.9%
LTR elements:								
Copia	13.8	9.9	12.6	8.8	10.6	8.0	8.6	7.4
	3.7%	2.9%	3.1%	2.6%	3.3%	2.6%	2.3%	2.2%
Gypsy	79.2	47.9	90.9	39.3	44.5	35.3	37.1	30.3
	21.1%	14.2%	22.1%	11.6%	14.0%	11.4%	9.9%	9.0%
**DNA transposons**								
TcMar-Stowaway	10.0	9.7	10.6	9.5	8.5	9.6	9.4	8.3
	2.7%	2.9%	2.6%	2.8%	2.7%	3.1%	2.5%	2.5%
PIF-Harbinger	10.8	10.7	11.4	10.4	9.3	10.3	10.1	9.1
	2.9%	3.2%	2.8%	3.1%	2.9%	3.3%	2.7%	2.7%
MULE-MuDR	14.9	12.0	14.9	11.4	11.0	10.5	10.7	9.6
	4.0%	3.6%	3.6%	3.4%	3.5%	3.4%	2.9%	2.8%
CMC-EnSpm	17.4	11.5	14.5	9.1	10.5	7.8	8.7	6.7
	4.7%	3.4%	3.5%	2.7%	3.3%	2.5%	2.3%	2.0%
hAT	2.6	1.9	2.2	2.2	2.0	1.9	1.8	6.7
	0.7%	0.6%	0.5%	0.7%	0.6%	0.6%	0.5%	2.0%
RC/Helitron	5.4	4.7	5.2	4.4	3.7	3.8	3.8	2.7
	1.4%	1.4%	1.3%	1.3%	1.2%	1.2%	1.0%	0.8%
**Total interspersed**	140.4	103.3	151.7	86.8	91.6	83.6	81.7	61.7
	37.5%	30.5%	35.5%	25.7%	28.9%	27.1%	21.9%	18.4%

[a] Coverage of TEs Mbp (mega base pairs).
[b] The proportion of TEs of the total genome size.
