# Supplementary material for: Domestication of rice has reduced the occurrence of transposable elements within gene coding regions
Source: BMC Genomics. 2017 Jan 9;18:55. doi: 10.1186/s12864-016-3454-z (PMC5223533; doi:10.1186/s12864-016-3454-z)
Supplement: Additional file 3: Table S3. — Transposon insertions in the Genomes, and in the gene regions. (PDF 30 kb) [file 12864_2016_3454_MOESM3_ESM.pdf]

Supplementary table S3. Transposon insertions in the Genomes, and in the gene regions

Repeat family	*O.sat*	*O.ruf*	*O.ind*	*O.niv*	*O.gla*	*O.bar*	*O.glu*	*O.mer*
**Retroelements**								
SINEs:	5245[a] (668[b])<br>12.7%[c]	5284 (1244)<br>23.5%	5769 (662)<br>11.5%	5166 (1238)<br>24.0%	4600 (489)<br>10.6%	5100 (1047)<br>20.5%	5058 (1250)<br>24.7%	4275 (1046)<br>24.5%
LINEs:	5441 (789)<br>14.5%	5685 (1463)<br>25.7%	6036 (941)<br>15.6%	5283 (1308)<br>24.8%	4883 (628)<br>12.9%	5233 (1144)<br>21.9%	5203 (1303)<br>25.0%	4584 (1166)<br>25.4%
LTR elements:								
Copia	7851 (683)<br>8.7%	9113 (1596)<br>17.5%	9708 (759)<br>7.8%	7337 (1236)<br>16.8%	7035 (845)<br>12.0%	7544 (1110)<br>14.7%	8065 (1439)<br>17.8%	6885 (1220)<br>17.7%
Gypsy	34349 (1788)<br>5.2%	46281 (5587)<br>12.1%	58516 (1877)<br>3.2%	30685 (3567)<br>11.6%	26089 (1577)<br>6.0%	26215 (2503)<br>9.5%	32691 (4660)<br>14.2%	27494 (2778)<br>10.1%
**DNA transposons**								
TcMar-Stowaway	51301 (8012)<br>15.6%	51277 (12798)<br>25.0%	55109 (7913)<br>14.4%	49820 (12783)<br>25.7%	45109 (6055)<br>13.4%	49723 (11180)<br>22.5%	49148 (13191)<br>26.8%	44932 (12313)<br>27.4%
PIF-Harbinger	49061 (4797)<br>9.8%	49954 (9267)<br>18.5%	53246 (4950)<br>9.3%	48286 (9440)<br>19.5%	43004 (3396)<br>7.9%	47715 (7414)<br>15.5%	46654 (9491)<br>20.3%	43573 (9107)<br>20.9%
MULE-MuDR	38559 (4789)<br>12.4%	40135 (9617)<br>24.0%	41725 (4948)<br>11.9%	36946 (8840)<br>23.9%	33905 (4156)<br>12.3%	35590 (7222)<br>20.3%	35659 (8913)<br>25.0%	31573 (7895)<br>25.0%
CMC-EnSpm	24648 (2372)<br>9.6%	26665 (4682)<br>17.6%	26834 (2454)<br>9.15%	22671 (4171)<br>18.4%	20407 (1872)<br>9.2%	19928 (3041)<br>15.3%	21478 (3990)<br>18.6%	19400 (3286)<br>16.9%
hAT	9786 (1199)<br>12.2%	10020 (2116)<br>21.1%	10442 (1066)<br>10.2%	9143 (1975)<br>21.6%	8079 (826)<br>10.2%	8563 (1572)<br>18.4%	8565 (1979)<br>23.1%	7770 (1747)<br>22.5%
RC/Helitron	9533 (1102)<br>11.6%	8395 (1746)<br>20.8%	9937 (982)<br>9.9%	8020 (1713)<br>21.4%	6317 (623)<br>9.9%	6214 (1073)<br>17.3%	6649 (1363)<br>20.5%	4454 (810)<br>18.2%
**Total interspersed**	257161 (28780)<br>11.2%	274960 (55084)<br>20.0%	300701 (29157)<br>9.7%	244092 (51021)<br>20.9%	218016 (22420)<br>10.3%	232531 (41383)<br>17.8%	239641 (52462)<br>21.9%	212245 (45592)<br>21.5%

[a] Copy No. of TEs in genome.
[b] Copy No. of TEs in genes.
[c] The percent of TEs in genes.
