# Supplementary figures and images for: Domestication of rice has reduced the occurrence of transposable elements within gene coding regions
Source: BMC Genomics. 2017 Jan 9;18:55. doi: 10.1186/s12864-016-3454-z (PMC5223533; doi:10.1186/s12864-016-3454-z)

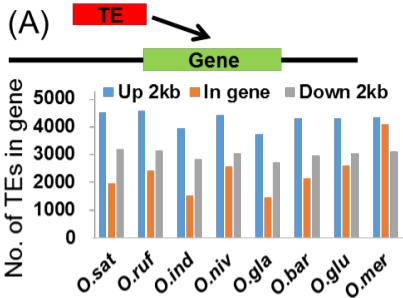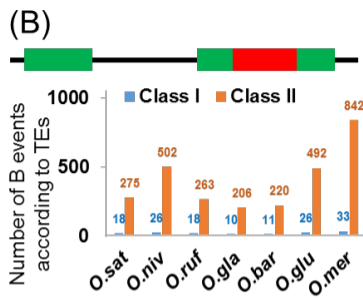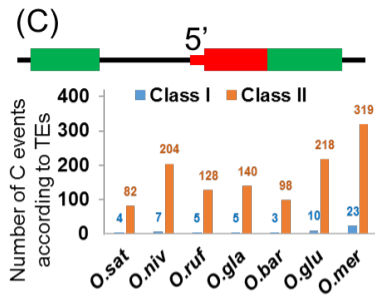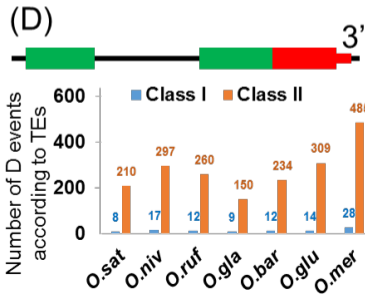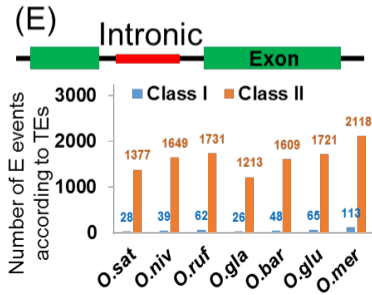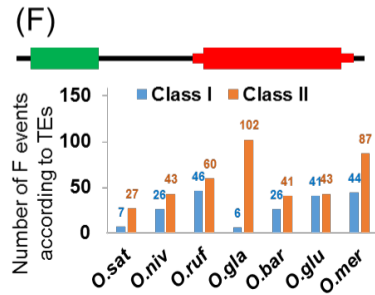

Supplement: Additional file 4: Figure S1. — TEs location in 2384 genes obtained from conserved synteny regions via Oryza transcriptome data. (PDF 133 kb) [file 12864_2016_3454_MOESM4_ESM.pdf]

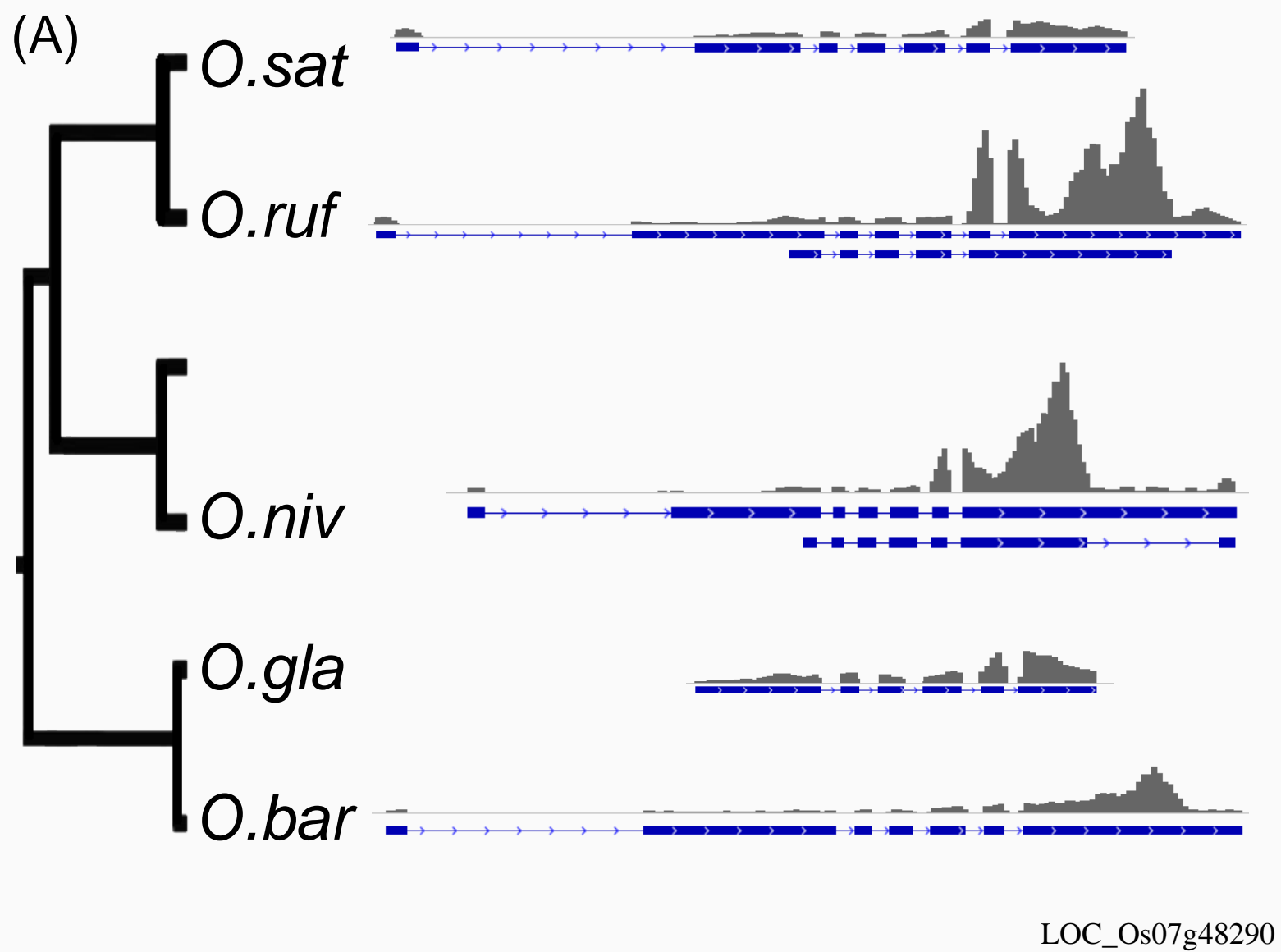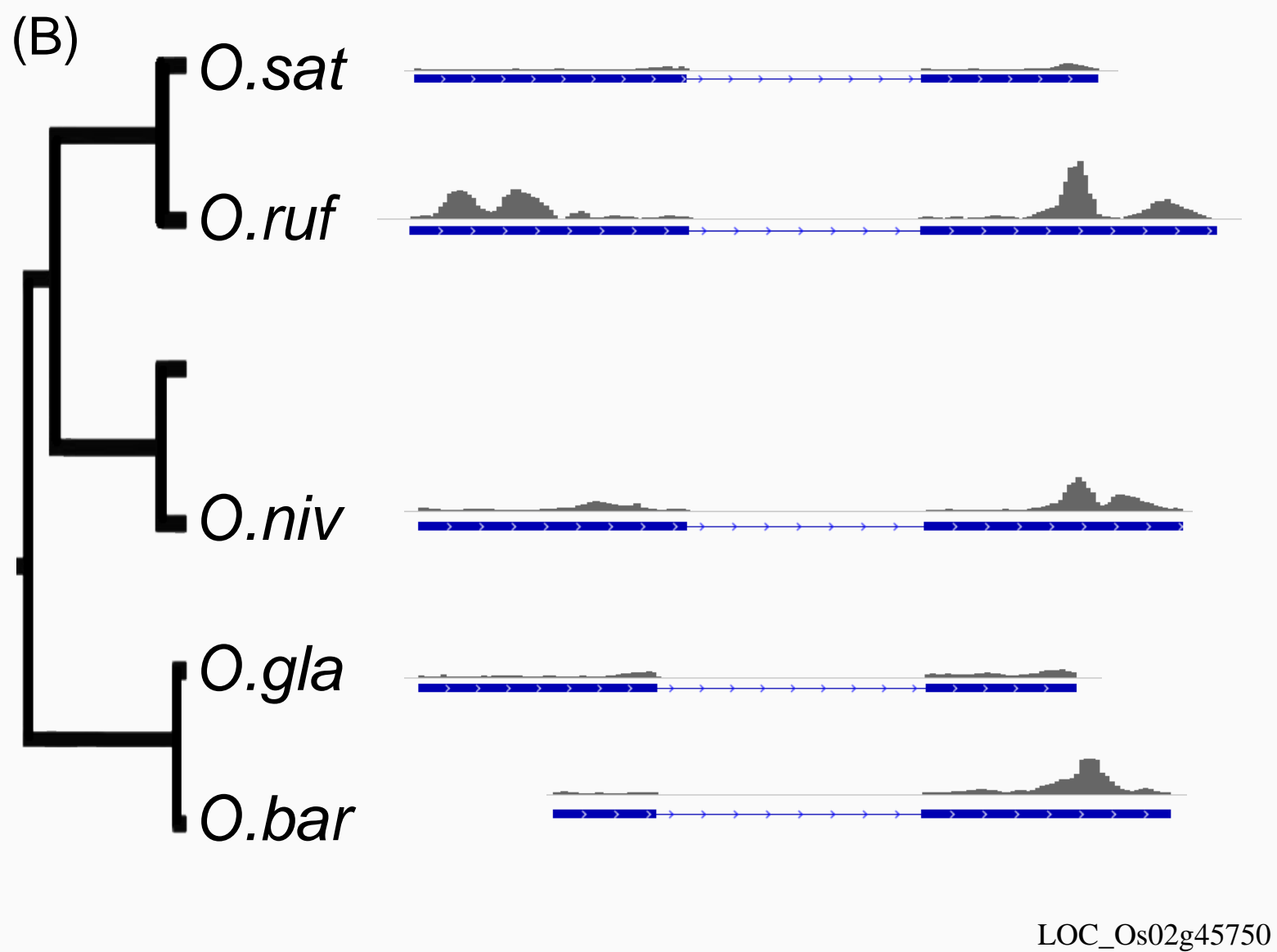

Supplement: Additional file 9: Figure S2. — The gene structures are supported by RNA-seq data. (A) LOC_Os07g48290 gene. (B) LOC_Os02g45750 gene. (PDF 27 kb) [file 12864_2016_3454_MOESM9_ESM.pdf]
