# Supplementary material for: Domestication of rice has reduced the occurrence of transposable elements within gene coding regions
Source: BMC Genomics. 2017 Jan 9;18:55. doi: 10.1186/s12864-016-3454-z (PMC5223533; doi:10.1186/s12864-016-3454-z)
Supplement: Additional file 5: Table S4. — Transposon insertions in the Genomes, and in the 0-2 kb regions upstream of a gene. (PDF 30 kb) [file 12864_2016_3454_MOESM5_ESM.pdf]

**Supplementary table S4. Transposon insertions in the Genomes, and in the 0-2kb regions upstream of ATG start codon of a gene.**

| Repeat family | *O.sat* | *O.ruf* | *O.ind* | *O.niv* | *O.gla* | *O.bar* | *O.glu* | *O.mer* |
|---|---|---|---|---|---|---|---|---|
| **Retroelements** | | | | | | | | |
| SINEs: | 5245[a] (1352[b]) 25.8%[c] | 5284 (1454) 27.5% | 5769 (1517) 26.3% | 5166 (1451) 28.1% | 4600 (1155) 25.1% | 5100 (1390) 27.2% | 5058 (1371) 27.1% | 4275 (1095) 25.6% |
| LINEs: | 5441 (910) 16.7% | 5685 (1011) 17.8% | 6036 (1117) 18.5% | 5283 (977) 18.5% | 4883 (806) 16.5% | 5233 (901) 17.2% | 5203 (1013) 19.5% | 4584 (809) 17.6% |
| LTR elements: | | | | | | | | |
| Copia | 7851 (647) 8.2% | 9113 (1023) 11.2% | 9708 (991) 10.2% | 7337 (893) 12.2% | 7035 (658) 9.3% | 7544 (912) 12.09% | 8065 (941) 11.7% | 6885 (737) 10.7% |
| Gypsy | 34349 (2009) 5.8% | 46281 (4319) 9.3% | 58516 (3229) 5.5% | 30685 (3146) 10.2% | 26089 (1968) 7.5% | 26215 (2645) 10.1% | 32691 (3609) 11.0% | 27494 (2505) 9.1% |
| **DNA transposons** | | | | | | | | |
| TcMar-Stowaway | 51301 (13714) 26.7% | 51277 (14084) 27.5% | 55109 (14789) 26.8% | 49820 (13846) 27.8% | 45109 (11877) 26.3% | 49723 (12182) 24.5% | 49148 (13421) 27.3% | 44932 (11033) 24.5% |
| PIF-Harbinger | 49061 (14937) 30.4% | 49954 (15426) 30.9% | 53246 (15949) 29.9% | 48286 (15197) 31.5% | 43004 (12503) 29.1% | 47715 (14559) 30.5% | 46654 (14301) 30.6% | 43573 (12177) 27.9% |
| MULE-MuDR | 38559 (9990) 25.9% | 40135 (11950) 29.8% | 41725 (11171) 26.8% | 36946 (11110) 30.1% | 33905 (9300) 27.4% | 35590 (10474) 29.4% | 35659 (10496) 29.4% | 31573 (8444) 26.7% |
| CMC-EnSpm | 24648 (3961) 16.1% | 26665 (4794) 18.0% | 26834 (4808) 17.9% | 22671 (4447) 19.6% | 20407 (3456) 16.9% | 19928 (3746) 18.8% | 21478 (4057) 18.9% | 19400 (3309) 17.1% |
| hAT | 9786 (2364) 24.2% | 10020 (2613) 26.1% | 10442 (2574) 24.6% | 9143 (2364) 25.9% | 8079 (1944) 24.1% | 8563 (2088) 24.4% | 8565 (2214) 25.8% | 7770 (1792) 23.1% |
| RC/Helitron | 9533 (1360) 14.3% | 8395 (1331) 15.8% | 9937 (1694) 17.0% | 8020 (1333) 16.6% | 6317 (1003) 15.9% | 6214 (965) 15.5% | 6649 (1074) 16.1% | 4454 (640) 14.4% |
| **Total interspersed** | 257161 (57101) 22.2% | 274960 (64093) 23.3% | 300701 (64010) 21.3% | 244092 (60669) 24.8% | 218016 (49715) 22.8% | 232531 (56567) 24.3% | 239641 (58237) 24.3% | 212245 (47133) 22.2% |

[a] Copy No. of TEs in genome.

[b] Copy No. of TEs upstream of genes.

[c] The percent of TEs upstream of genes.
