# Supplementary material for: Domestication of rice has reduced the occurrence of transposable elements within gene coding regions
Source: BMC Genomics. 2017 Jan 9;18:55. doi: 10.1186/s12864-016-3454-z (PMC5223533; doi:10.1186/s12864-016-3454-z)
Supplement: Additional file 6: Table S5. — Transposon insertions in Genome and 0-2 kb downstream of a gene. (PDF 31 kb) [file 12864_2016_3454_MOESM6_ESM.pdf]

Supplementary table S5. Transposon insertions in Genome and 0-2kb downstream of the stop codon of a gene.

Repeat family	*O.sat*	*O.ruf*	*O.ind*	*O.niv*	*O.gla*	*O.bar*	*O.glu*	*O.mer*
**Retroelements**								
SINEs:	5245[a] (1134[b]) 21.6%[c]	5284 (1251) 23.7%	5769 (1315) 22.8%	5166 (1175) 22.7%	4600 (985) 21.4%	5100 (1110) 21.8%	5058 (1175) 23.2%	4275 (908) 21.2%
LINEs:	5441 (946) 17.4%	5685 (1042) 18.3%	6036 (1158) 19.2%	5283 (966) 18.3%	4883 (888) 18.2%	5233 (945) 18.1%	5203 (947) 18.2%	4584 (806) 17.6%
LTR elements:								
Copia	7851 (696) 8.9%	9113 (1028) 11.3%	9708 (1037) 10.7%	7337 (929) 12.7%	7035 (703) 10.0%	7544 (950) 12.6%	8065 (982) 12.2%	6885 (811) 11.8%
Gypsy	34349 (1891) 5.5%	46281 (3815) 8.2%	58516 (3006) 5.1%	30685 (2822) 9.2%	26089 (1820) 7.0%	26215 (2492) 9.5%	32691 (3353) 10.3%	27494 (2309) 8.4%
**DNA transposons**								
TcMar-Stowaway	51301 (11765) 22.9%	51277 (11746) 22.9%	55109 (12625) 22.9%	49820 (11285) 22.6%	45109 (10120) 22.4%	49723 (10864) 21.8%	49148 (11140) 22.7%	44932 (9360) 20.8%
PIF-Harbinger	49061 (11583) 23.6%	49954 (11543) 23.1%	53246 (11757) 22.1%	48286 (11226) 23.2%	43004 (9447) 22.0%	47715 (10466) 21.9%	46654 (10744) 23.0%	43573 (9380) 21.5%
MULE-MuDR	38559 (7775) 20.2%	40135 (9059) 22.6%	41725 (8786) 21.1%	36946 (8589) 23.2%	33905 (6918) 20.4%	35590 (7929) 22.3%	35659 (7987) 22.4%	31573 (6705) 21.2%
CMC-EnSpm	24648 (3507) 14.2%	26665 (4089) 15.3%	26834 (4238) 15.8%	22671 (3865) 17.0%	20407 (3071) 15.0%	19928 (3400) 17.1%	21478 (3611) 16.8%	19400 (2955) 15.2%
hAT	9786 (2125) 21.7%	10020 (2204) 22.0%	10442 (2279) 21.8%	9143 (1998) 21.8%	8079 (1713) 21.2%	8563 (1877) 21.9%	8565 (1898) 22.2%	7770 (1548) 19.9%
RC/Helitron	9533 (1207) 12.7%	8395 (1167) 13.9%	9937 (1525) 15.3%	8020 (1204) 15.0%	6317 (895) 14.2%	6214 (864) 13.9%	6649 (956) 14.4%	4454 (604) 13.6%
**Total interspersed**	257161 (47630) 18.5%	274960 (52074) 18.9%	300701 (53063) 17.6%	244092 (48942) 20.0%	218016 (40763) 18.7%	232531 (45529) 19.6%	239641 (47550) 19.8%	212245 (39186) 18.5%

[a] Copy No. of TE Insertions in genome.

[b] Copy No. of TE Insertions downstream of genes.

[c] The percent of TEs insertions downstream of genes.
