# Supplementary material for: Domestication of rice has reduced the occurrence of transposable elements within gene coding regions
Source: BMC Genomics. 2017 Jan 9;18:55. doi: 10.1186/s12864-016-3454-z (PMC5223533; doi:10.1186/s12864-016-3454-z)
Supplement: Additional file 7: Table S6. — Statistical table of transposon insertions in Genome and gene. (PDF 252 kb) [file 12864_2016_3454_MOESM7_ESM.pdf]

Supplementary table S6. Statistical table of transposon insertions in Genome and gene.

Location	*O.sat* VS *O.ruf*		*O.ind* VS *O.niv*		*O.sat* VS *O.niv*		*O.ind* VS *O.ruf*		*O.gla* VS *O.bar*		*O.ruf* VS *O.niv*	
	χ^2	P-value	χ^2	P-value	χ^2	P-value	χ^2	P-value	χ^2	P-value	χ^2	P-value
**In gene:**												
SINEs	206.12[a]	3.2E-45	295.25	3.6E-66	218.65	1.8E-49	280.60	5.6E-63	177.08	2.1E-40	0.23	**0.6288**
LINEs	216.62	5.0E-49	148.18	4.3E-34	178.64	9.6E-41	184.18	5.9E-42	141.00	1.6E-32	1.33	**0.2489**
Copia	280.97	4.6E-63	328.69	1.9E-73	227.29	2.3E-51	402.73	1.4E-89	22.66	1.9E-06	1.22	**0.2687**
Gypsy	1117.80	4.5E-245	2487.00	0	882.96	5.0E-194	3068.20	0	222.65	2.4E-50	3.48	**0.0621**
TcMar-Stowaway	1383.10	1.0E-302	2109.30	0	1559.30	0	1902.90	0	1305.30	7.9E-286	6.51	**0.0107**
PIF-Harbinger	1562.70	0	2187.80	0	1860.60	0	1857.60	0	1257.50	1.9E-275	15.83	6.9E-05
MULE-MuDR	1750.90	0	1973.60	0	1688.10	0	2047.90	0	818.04	6.4E-180	0.01	**0.9166**
CMC-EnSpm	679.50	8.6E-150	906.84	3.2E-199	762.30	8.5E-168	818.30	5.7E-180	348.62	8.5E-78	5.81	**0.016**
hAT	278.59	1.5E-62	481.49	1.0E-106	295.36	3.4E-66	462.54	1.3E-102	222.35	2.8E-50	0.64	**0.4248**
RC/Helitron	284.42	8.2E-64	457.39	1.8E-101	309.89	2.3E-69	427.21	6.6E-95	146.15	1.2E-33	0.74	**0.3888**
Total	7824.40	0	13479	0	8821.60	0	12285	0	5224.40	0	59.97	9.6E-15
**0-2kb upstream:**												
SINEs	3.99	**0.0458**	4.33	**0.03735**	6.95	0.0084	2.03	**0.1541**	5.65	**0.0175**	0.40	**0.5294**
LINEs	2.11	**0.1465**	7.9E-30	**1.00**	5.66	**0.01736**	0.98	**0.3224**	0.86	**0.3534**	0.88	**0.3476**
Copia	42.00	9.1E-11	16.18	5.8E-150	63.87	1.3E-15	4.99	**0.02552**	28.07	1.2E-07	3.44	**0.0637**
Gypsy	330.30	8.3E-74	679.21	9.9E-16	430.05	1.6E-95	561.85	3.3E-124	105.10	1.2E-24	17.74	2.5E-05
TcMar-Stowaway	6.96	0.00835	12.01	0.00053	14.26	0.00016	5.31	**0.02121**	41.73	1.0E-10	1.32	**0.25**
PIF-Harbinger	2.18	**0.1399**	27.42	1.6E-07	11.96	0.00054	10.42	0.00124	22.29	2.3E-06	3.99	**0.0458**
MULE-MuDR	146.00	1.3E-33	104.83	1.3E-24	162.15	3.8E-37	90.79	1.6E-21	34.02	5.5E-09	0.79	**0.3735**
CMC-EnSpm	32.829	1.0E-08	23.19	1.5E-06	101.33	7.8E-24	0.03	**0.8629**	23.71	1.1E-06	21.46	3.6E-06
hAT	9.61	0.00194	3.69	**0.05465**	7.19	0.00733	5.43	**0.01979**	0.22	**0.6413**	0.11	**0.7387**
RC/Helitron	8.70	0.00317	0.55	**0.46**	18.41	1.8E-05	4.61	**0.03174**	0.26	**0.6092**	1.72	**0.1903**
Total	92.28	7.5E-22	971.70	2.6E-213	489.32	2.0E-108	339.63	7.7E-76	144.76	2.4E-33	168.96	1.2E-38
**0-2kb downstream:**												
SINEs	6.23	**0.0126**	1.5E-03	**0.9692**	1.84	**0.1748**	1.15	**0.2831**	0.16	**0.6924**	1.22	**0.27**
LINEs	1.62	**0.2032**	1.44	**0.2305**	1.42	**0.234**	1.35	**0.2451**	0.02	**0.8887**	1.2E-03	**0.9724**
Copia	26.69	2.4E-07	15.86	6.8E-05	56.82	4.8E-14	1.66	**0.1971**	24.22	8.6E-07	7.27	0.007
Gypsy	224.33	1.0E-50	542.58	5.2E-120	328.00	2.6E-73	409.20	5.5E-91	110.30	8.4E-26	21.17	4.2E-06
TcMar-Stowaway	8.6E-03	**0.926**	0.97	**0.3241**	1.12	**0.2891**	1.4E-06	**0.9991**	4.67	**0.03067**	0.92	**0.3368**
PIF-Harbinger	3.46	**0.0629**	19.68	9.2E-06	1.74	**0.1869**	15.49	8.3E-05	0.01	**0.91**	0.27	**0.604**
MULE-MuDR	67.64	2.0E-16	54.51	1.5E-13	105.50	9.5E-25	27.43	1.6E-07	36.21	1.8E-09	4.94	**0.0262**
CMC-EnSpm	12.34	0.00044	14.04	0.00018	71.18	3.3E-17	2.11	**0.1467**	30.17	4.0E-08	26.48	2.7E-07
hAT	0.21	**0.6443**	8.5E-04	**0.9768**	0.05	**0.8318**	0.08	**0.7809**	1.22	**0.2693**	0.05	**0.8244**
RC/Helitron	5.87	**0.01544**	0.36	**0.549**	20.12	7.3E-06	7.48	0.00626	0.16	**0.6894**	4.01	**0.0452**
Total	15.17	9.8E-05	511.58	2.9E-113	188.16	8.0E-43	160.60	8.4E-37	56.54	5.5E-14	101.92	5.8E-24

[a] Take the case of SINEs of TEs in gene; we calculated the χ^2 by "2-sample test for equality of proportions with continuity correction". The R code is prop.test(c(668,1244),c(5245,5284)).
